# Supplementary material for: Content-rich biological network constructed by mining PubMed abstracts
Source: BMC Bioinformatics. 2004 Oct 8;5:147. doi: 10.1186/1471-2105-5-147 (PMC528731; doi:10.1186/1471-2105-5-147)
Supplement: Additional File 2 — The original results of the above study (non-essential files are deleted to keep the file size under the limit set by BMC bioinformatics). [file 1471-2105-5-147-S2.bz2 › chilibotAdditionalFile2/dip30/727ID9822594E18076/html/HLYB_HLYA.html]

 


 **HLYB** and **HLYA** 
  
Found 91 abstracts in PubMed, retrieved 30.  
 

 What does Google say? 
 PDF only 
| .edu only 

---

**Interactive relationship** (e.g. stimulation, inhibition, etc)

**Neutral relationship**- Our data suggest that TnhlyAs generated hemolysin  [ **HLYA** ]  fusion proteins are secreted efficiently by the  **HlyB**  HlyD TolC hemolysin  [ **HLYA** ]  secretion machinery.  Ref: 9711856 FEMS Microbiol Lett, 1998
- Preliminary sequence analysis indicated that another open reading frame homolog to the  **hlyB**  gene is located close to the 3 end of EHEC  **hlyA** .  Ref: 7868227 Infect Immun, 1995
- The secretion of the 107 kDa hemolysin  [ **HLYA** ]  A  **HlyA**  from Escherichia coli is mediated by membrane proteins hemolysin  [ **HLYA** ]  B  **HlyB**  and hemolysin  [ **HLYA** ]  D HlyD.  Ref: 7703231 Biochemistry, 1995
- These lie downstream of  **hlyA**  and  **hlyB** , which encode the El Tor hemolysin  [ **HLYA** ]  and methyl accepting chemotactic factor, respectively.  Ref: 9371455 J Bacteriol, 1997
- The mutant  **HlyB**  HlyDDelta45 inner membrane complex engaged the hemolysin  [ **HLYA** ]  substrate, but this substrate engaged complex failed to trigger recruitment of TolC.  Ref: 11676535 J Mol Biol, 2001
- Point mutants in  **HlyB**  compensate for a deletion in the predicted amphiphilic helix region of the  **HlyA**  signal.  Ref: 7782350 J Biol Chem, 1995
- Identification and preliminary characterization of temperature sensitive mutations affecting  **HlyB** , the translocator required for the secretion of haemolysin  **HlyA**  from Escherichia coli.  Ref: 7808392 Mol Gen Genet, 1994
- Selection for transport competence of C terminal polypeptides derived from Escherichia coli hemolysin  [ **HLYA** ]  the shortest peptide capable of autonomous  **HlyB**  HlyD dependent secretion comprises the C terminal 62 amino acids of  **HlyA** .  Ref: 7531275 Mol Gen Genet, 1994
- We show that an scFv  **HlyA**  hybrid secreted by Hly type I transporter TolC,  **HlyB** , HlyD is accumulated in the extracellular medium with the disulphide bonds correctly formed.  Ref: 11309117 Mol Microbiol, 2001
- These suppressor mutations demonstrated a direct interaction between  **HlyA**  and  **HlyB** .  Ref: 7782350 J Biol Chem, 1995
- This set of  **HlyB**  mutants identifies further sites in  **HlyB**  that modulate substrate specificity but display allele specific effects on a range of  **HlyA**  signal mutants.  Ref: 7782350 J Biol Chem, 1995
- Mutations in  **hlyB**  affecting secretion could be assigned to two classes those that restore the level of vancomycin resistance to that of E. coli not secreting  **HlyA**  and those that still confer hypersensitivity to the drug in the presence of  **HlyA** .  Ref: 7808392 Mol Gen Genet, 1994
- The system which consists of the two specific membrane proteins  **HlyB**  and HlyD and the outer membrane protein TolC, recognizes on  **HlyA**  a C terminally located signal sequence of about 60 amino acids.  Ref: 9382730 Behring Inst Mitt, 1997
- The genetic determinants for a fusion of its B subunit StxB, which mediates toxin binding to target cells, with the COOH terminus of Escherichia coli hemolysin  [ **HLYA** ]  A Stx  **HlyA**  has been combined with the determinants of the accessory translocator proteins  **HlyB**  and HlyD in a Notl cassette.  Ref: 8905616 Microb Pathog, 1996
- The export of Escherichia coli hemolysin  [ **HLYA** ]  across the cytoplasmic and the outer membranes requires the COOH terminal signal sequence of  **HlyA** , the two specific translocator proteins  **HlyB**  and HlyD, and the outer membrane protein TolC.  Ref: 9630987 Nat Biotechnol, 1996
- Secretion of Escherichia coli hemolysin  [ **HLYA** ]  is mediated by a sec independent pathway which requires the products of at least three genes,  **hlyB** , hlyD and tolC.  Ref: 7816028 Mol Gen Genet, 1994
- We engineered plasmid pETR14, containing the  **hlyB**  and hlyD genes of the Escherichia coli hemolysin  [ **HLYA** ]  operon, to express a fusion protein containing 720 amino acid residues from the nontoxic, receptor binding, carboxy terminus of C. difficile toxin A and the secretion signal of E. coli hemolysin  [ **HLYA** ]  A.  Ref: 9199470 Infect Immun, 1997

**Non-interactive relationship** (e.g. studied together, co-existance, homology, etc.)

- This PagC HlyAs fusion protein was expressed and efficiently secreted into the culture supernatant by E. coli upon complementation with the hemolysin  [ **HLYA** ]  secretion proteins  **HlyB**  and HlyD.  Ref: 8766698 Appl Microbiol Biotechnol, 1996
- Mutant  **HlyB**  that bound, but did not hydrolyse ATP, supported IM complex assembly, substrate recruitment and bridging, but  **HlyA**  stalled in the channel.  Ref: 9822594 EMBO J, 1998
- In the present study, we selected  **HlyB**  alleles that complemented an internal deletion of 29 amino acids in  **HlyA**  containing a predicted amphiphilic helix region immediately upstream from the previous deletion.  Ref: 7782350 J Biol Chem, 1995
- Our data suggest that all beta Gal HlyAs fusion proteins generated are secreted, albeit with varying efficiencies, by the  **HlyB**  HlyD TolC hemolysin  [ **HLYA** ]  secretion machinery under Sec proficient conditions.  Ref: 8842146 Mol Gen Genet, 1996
- However, hemolytic activity was found in the broth culture supernatant after transforming EDL 933 with the recombinant plasmid pRSC6 carrying the  **hlyB**  and hlyD genes from the E. coli alpha hemolysin  [ **HLYA** ]  operon.  Ref: 7868227 Infect Immun, 1995
- The deduced amino acid sequence showed significant homology to the C terminal region of  **HlyB**  31.1% identical residues, a protein located in the Escherichia coli alpha hemolysin  [ **HLYA** ]  operon that is a representative bacterial ATP binding cassette export protein.  Ref: 9401046 J Bacteriol, 1997
- The presence of fragments of genomes  **hlyA** ,  **hlyB** , papAH, papC, sfaG, sfaA and kps MT, associated with the pathogenicity islands of Escherichia coli, in clinical strains of other genera of the family Enterobacteriaceae, has been experimentally evaluated with the use of PCR.  Ref: 12506618 Zh Mikrobiol Epidemiol Immunobiol< MedlineTA>Zh Mikrobiol Epidemiol Immunobiol, 1997
- Previously, it was shown that point mutants in the transmembrane domain of the transporter  **HlyB**  could partially correct the transport defect caused by a deletion of the C terminal 29 amino acids of  **HlyA** .  Ref: 7782350 J Biol Chem, 1995
- The structure and function of the components of the  **HlyA**  secretion apparatus,  **HlyB** , HlyD and TolC, have been studied in great detail.  Ref: 11755084 Trends Microbiol, 2002
- These effects are only observed in the presence of functional  **HlyB**  and TolC, the two other components of the hemolysin  [ **HLYA** ]  secretion machinery.  Ref: 9393456 Mol Gen Genet, 1997
- We found the CVD 419 probe to be identical to the 3.4 kb HindIII fragment of plasmid pEO40 carrying most of the EHEC  **hlyA**  gene and a part of the putative EHEC  **hlyB**  gene.  Ref: 7868227 Infect Immun, 1995
- Taking these results together, we propose a model for an early stage of initiation of secretion in vivo, in which the NBD of  **HlyB** , specifically recognizes the C terminus of the transport substrate,  **HlyA** , and where secretion is initiated by subsequent displacement of  **HlyA**  from  **HlyB**  by ATP.  Ref: 12662939 J Mol Biol, 2003
- To this end, Salmonella typhimurium SL7207 was used as carrier for secreted p60  **HlyA**  fusion protein by Escherichia coli  **HlyB**  and HlyD transport proteins.  Ref: 7729919 Infect Immun, 1995
- We have purified the nucleotide binding domain of  **HlyB**   **HlyB**  NBD and a C terminal 23kDa fragment of  **HlyA**  plus the His tag HlyA1, which contains the secretion sequence.  Ref: 12662939 J Mol Biol, 2003
- We have developed an export cloning system that is composed of two vectors one in which the fusion of the desired gene with the 3 end of  **hlyA**  is generated, and a second in which the sequences containing the fusion are combined with the accessory genes  **hlyB**  and hlyD, thereby reconstructing the natural organization of the hly locus.  Ref: 9630987 Nat Biotechnol, 1996
- This 70 amino acid peptide contains little primary sequence similarity to the  **HlyA**  signal sequence, and we have hypothesized that these signal sequences assume a similar higher order structure which is recognized by the  **HlyB**  D transporter.  Ref: 7703231 Biochemistry, 1995
- The toxin  **HlyA**  is exported from Escherichia coli, without a periplasmic intermediate, by a type I system comprising an energized inner membrane IM translocase of two proteins, HlyD and the traffic ATPase  **HlyB** , and the outer membrane OM porin like TolC.  Ref: 9822594 EMBO J, 1998
- Despite the Ts character of the Gly10 substitution, a derivative of  **HlyB** , in which the first 25 amino acids were replaced by 21 amino acids of the lambda Cro protein, was still active in secretion of  **HlyA** .  Ref: 7808392 Mol Gen Genet, 1994
